# *In silico* polymorphism analysis for the development of simple sequence repeat and transposon markers and construction of linkage map in cultivated peanut

**DOI:** 10.1186/1471-2229-12-80

**Published:** 2012-06-06

**Authors:** Kenta Shirasawa, Padmalatha Koilkonda, Koh Aoki, Hideki Hirakawa, Satoshi Tabata, Manabu Watanabe, Makoto Hasegawa, Hiroyuki Kiyoshima, Shigeru Suzuki, Chikara Kuwata, Yoshiki Naito, Tsutomu Kuboyama, Akihiro Nakaya, Shigemi Sasamoto, Akiko Watanabe, Midori Kato, Kumiko Kawashima, Yoshie Kishida, Mitsuyo Kohara, Atsushi Kurabayashi, Chika Takahashi, Hisano Tsuruoka, Tsuyuko Wada, Sachiko Isobe

**Affiliations:** 1 Kazusa DNA Research Institute, 2-6-7 Kazusa-Kamatari, Kisarazu, Chiba, 292-0818, Japan; 2 Chiba Prefectural Agriculture and Forestry Research Center, 808 Daizennocho, Midori, Chiba, 266-0006, Japan; 3 Mitsubishi Chemical Medience Corporation, 4-25-11 Azusawa, Itabashi, Tokyo, 174-0051, Japan; 4 College of Agriculture, Ibaraki University, 3-21-1 Chuo, Ami, Ibaraki, 300-0393, Japan; 5 Center for Transdisciplinary Research, Niigata University, 1-757 Asahimachidori, Chuo, Niigata, 951-8585, Japan; 6 Graduate School of Life & Environmental Sciences, Osaka Prefecture University, 1-1 Gakuencho, Naka, Sakai, Osaka, 599-8531, Japan

**Keywords:** DNA marker, Genetic linkage map, Peanut (*Arachis hypogaea*), QTL analysis, Ratio of oleic/linoleic acid (O/L ratio)

## Abstract

**Background:**

Peanut (*Arachis hypogaea*) is an autogamous allotetraploid legume (2n = 4x = 40) that is widely cultivated as a food and oil crop. More than 6,000 DNA markers have been developed in *Arachis* spp., but high-density linkage maps useful for genetics, genomics, and breeding have not been constructed due to extremely low genetic diversity. Polymorphic marker loci are useful for the construction of such high-density linkage maps. The present study used *in silico* analysis to develop simple sequence repeat-based and transposon-based markers.

**Results:**

The use of *in silico* analysis increased the efficiency of polymorphic marker development by more than 3-fold. In total, 926 (34.2%) of 2,702 markers showed polymorphisms between parental lines of the mapping population. Linkage analysis of the 926 markers along with 253 polymorphic markers selected from 4,449 published markers generated 21 linkage groups covering 2,166.4 cM with 1,114 loci. Based on the map thus produced, 23 quantitative trait loci (QTLs) for 15 agronomical traits were detected. Another linkage map with 326 loci was also constructed and revealed a relationship between the genotypes of the *FAD2* genes and the ratio of oleic/linoleic acid in peanut seed.

**Conclusions:**

*In silico* analysis of polymorphisms increased the efficiency of polymorphic marker development, and contributed to the construction of high-density linkage maps in cultivated peanut. The resultant maps were applicable to QTL analysis. Marker subsets and linkage maps developed in this study should be useful for genetics, genomics, and breeding in *Arachis*. The data are available at the Kazusa DNA Marker Database (http://marker.kazusa.or.jp).

## Background

Peanut (*Arachis hypogaea*) is an autogamous allotetraploid legume (2n = 4x = 40) composed of A and B genomes that are derived from two diploids, most likely *A. duranensis* (A genome) and *A. ipaënsis* (B genome). On the basis of branching habit, the presence/absence of flowers on the main stem, alternate *vs.* sequential branching, fruit and seed traits, and maturity, *A. hypogaea* has been categorized into two subspecies: *hypogaea* and *fastigiata*; six botanical varieties: *hypogaeahirsutafastigiatavulgarisaequatoriana*, and *peruviana*; and four agronomic types: Virginia, Spanish, Valencia, and Southeast-runner [1,2]. As the nuclear DNA content in peanut is calculated to be 5.914 pg/2 C [3], the genome size is estimated to be approximately 2.8 Gb based on an assumption that 1 pg of DNA is equivalent to 980 Mb [4]. Because of its allotetraploidy and large genome size, genomic study in the peanut has lagged far behind that of other legumes, such as *Lotus japonicus*[5], *Glycine max*[6], *Medicago truncatula*[7], and *Cajanus cajan*[8]. In addition, low genetic diversity within the species has inhibited the advance of genetic linkage map construction. In autogamous species, the genetic diversity of polyploids is generally more narrow than that of their diploid progenitors due to bottleneck effects, and this results in few alleles having been transferred from diploid progenitors to their polyploid descendents [9]. Moreover, genetic diversity is affected by the history of polyploidization. Tetraploid peanut is thought to have arisen approximately 3,500 years ago [10], and its short history has been considered a source of lower levels of polymorphism compared with diploid *Arachis* species [11].

At present, more than 6,000 DNA markers have been developed in *Arachis* spp. [12], *e.g.*, restriction fragment length polymorphism [13,14] and simple sequence repeats (SSRs) [11,15-21]. Linkage maps were constructed in wild diploids [14,19,22,23], cultivated species [21,24-26], and artificial amphidiploids derived from wild diploids [13,27]. Integrated maps based on cultivated peanuts were also constructed [28-31]. However, the peanut linkage map has not yet covered all of the chromosomes of the haploid genome (n = 20). Although extremely low genetic diversity has inhibited the construction of high-density genetic linkage maps in peanut, increasing the number of polymorphic markers is crucial for the saturation of linkage maps in peanut.

In general, SSR markers have been developed from randomly collected sequence data of complementary DNAs (cDNAs) [11,15], SSR-enriched genomic DNA libraries [16-20], and BAC-ends [21]. Primers were designed based on flanking regions of identified SSRs of the obtained sequences, and then polymorphism of the targeted SSRs was investigated using gel or capillary electrophoresis of DNA amplified by PCR. However, since the degree of polymorphism of the markers depends on the genetic diversity of the germplasm, experimental analysis requires considerable cost, time, and labor to develop a large number of polymorphic markers in species having low genetic diversity. Therefore, this strategy is not effective for the large-scale development of polymorphic DNA markers in closely-related lines. For the development of single nucleotide polymorphism (SNP) markers, on the other hand, *in silico* polymorphism analysis, *i.e.*, comparison of genomic or cDNA sequences derived from more than two lines, is often performed before synthesizing primers of DNA markers for lab validation. To our knowledge, this approach has been limited, *e.g.*, polySSR [32] and SSRpoly [33]. However, we consider that *in silico* polymorphism analysis prior to primer synthesis is also effective for SSR and other types of DNA marker development.

Recently, we have developed a total of 504 *AhMITE1* transposon markers in peanut [34]. The percentage of the transposon markers that were polymorphic between the two peanut lines was 22.0%, which was higher than that of the SSR markers [11,35]. This result suggested that transposon markers, like SSR markers, represent potent, co-dominant, and PCR-based markers.

Peanut is widely cultivated in Asia, Africa, America, and Australia as a food and oil crop. Peanut breeding has achieved a rise in productivity by increasing the size and number of seeds, and by enhancing resistance to biotic and abiotic stresses [36]. Breeding has been mostly performed by conventional methods, *e.g.*, a combination of crossing, phenotypic selections, and homogenization. In conventional breeding, large sizes of breeding populations are required, especially for the selection of recessive traits, because single gene mutations often do not confer phenotypic variation in peanut due to functional complementation by homoeologous genes. Thus, molecular breeding with marker-assisted selection has great promise and may lead to remarkable advances in peanut breeding. While quantitative trait locus (QTL) analysis is an effective method for identification of DNA markers linked to agronomically important traits, few QTL studies have been conducted due to the lack of high-density linkage maps in peanut [25,28,30,31].

Because peanut is used as an oil crop, seed quality is also an important breeding objective. The major components of peanut oil are linoleic acid and oleic acid, consisting of 36-67% and 15-43% of total oil, respectively, in normal cultivars [37]. Whereas normal cultivars have a ratio of oleic to linoleic acid (O/L ratio) of about 1:4, the ratio can reach as high as 1:30 or 1:40 in high-O/L ratio cultivars [38]. Oleic acid is a monounsaturated fatty acid, whereas linoleic acid is a polyunsaturated fatty acid. Therefore oleic acid is less oxidized than linoleic acid, and it is considered that oleic acid is better for health and storage quality [39]. In high-O/L ratio plants, oleic acid is synthesized from stearic acid and is converted into linoleic acid by two fatty acid desaturases encoded by *SAD* and *FAD2*[40]. The selection of mutated alleles of *FAD2*, which is associated with oleic-acid content in seeds [41-43], is a straightforward strategy to efficiently generate high-oleic acid crops. In peanut, *ahFAD2A* and *ahFAD2B* have been identified on the A and B genomes, respectively, and the mutant alleles are reported to confer a high O/L ratio [44-49].

In this study, we investigated whether *in silico* polymorphism analysis could increase the efficiency of development of polymorphic SSR and transposon markers. First, genomic sequences covering SSR and transposon-inserted regions derived from two peanut lines were compared *in silico* to identify candidate polymorphic regions. Then, the candidate polymorphic regions were subjected to lab validation. High-density genetic linkage maps were constructed using the developed polymorphic markers along with published markers. The developed linkage maps demonstrated their applicability to molecular breeding through QTL mapping for agronomical traits and *FAD2* gene mapping for the development of varieties with high O/L ratios.

## Methods

### Plant materials and DNA extraction

Two F_2_ mapping populations, *i.e.*, SKF2 and NYF2, were used for the construction of linkage maps. The SKF2 (n = 94) was generated from a cross between two lines belonging to different agronomic and morphological types, *i.e.*, a Virginia type, ‘Satonoka’, and a Spanish type, ‘Kintoki’. The SKF2 population is expected to generate a larger number of polymorphic markers for construction of a high-density linkage map. It has also been used for the identification of 15 agronomic trait loci. The NYF2 (n = 186) is a breeding population derived from a cross between a Virginia type, ‘YI-0311’, which is also considered as a Southeast-runner type, and another Virginia type, ‘Nakateyutaka’. The former is a breeding line showing a high O/L ratio in seeds, and the latter is a leading cultivar in Japan with a normal O/L ratio. This mapping population was used for the identification of linkages between genotypes of *FAD2* genes and O/L ratio in seeds. Genomic DNA from each line was extracted using the DNeasy Plant Mini kit (Qiagen, Germany).

### Development of SSR markers by *in silico* polymorphism analysis

To develop SSR markers, two SSR-enriched genomic libraries were constructed, as described by Nunome *et al.*[50]. While the first library was generated from a single line, ‘Satonoka’, the second library was developed using two lines, ‘Satonoka’ and ‘Kintoki’, for *in silico* polymorphism analysis. Both libraries were constructed with biotinylated oligo probes of (AC)_12_ and (CT)_12_. Sequencing analysis of all libraries and primer design for the first library were performed as described by Sraphet *et al.*[51]. For the second library, primers were designed based only on flanking sequences of polymorphic SSRs between ‘Satonoka’ and ‘Kintoki’, identified by *in silico* analysis as described below. The SSR motifs in the sequences were identified by the Fuzznuc tool from EMBOSS, version 6.1.0 [52], and the sequences with SSR motifs were assembled by the CAP3 program with parameters set to require 95% identify to be considered overlapping (−p 95) [53]. Of the obtained assemblies, contigs comprising sequences with different lengths of the same SSR motifs (different numbers of the same repeated sequence) between two lines were selected as polymorphic SSR candidates for primer design. All of the designed genomic SSR markers were designated as AHGS (*rachisypogaea*genomic SSR) markers.

### Development of transposon markers through *in silico* polymorphism analysis

Shirasawa *et al.*[34] reported that insertion sites in the peanut genome for the transposon *AhMITE1* differ among cultivars. They also demonstrated that the insertional polymorphisms of transposon elements can be used as DNA markers by developing 504 polymorphic markers derived from transposon-enriched genomic libraries. To develop additional transposon markers, transposon-enriched genomic libraries were constructed from ‘Satonoka’ and ‘Kintoki’, and sequences were obtained as previously described [34]. The individual sequences were assembled using the CAP3 program with default parameters [53]. Contigs and singlets having *AhMITE1* sequences only on one cultivar (either ‘Satonoka’ or ‘Kintoki’, but not both) were selected as candidate polymorphic sequences. Using the PRIMER3 program [54], primers were designed on both flanking sequences of *AhMITE1*, or on one of the flanking sequences and an internal sequence of *AhMITE1* in cases lacking either flanking sequence.

Additionally, the flanking sequences of the *AhMITE1* transposons were cloned using inverse PCR. Genomic DNA fragments digested by *Mbo*I, *Mse*I, or *Xsp*I were self-ligated using T4 DNA ligase (Promega, USA) and used as PCR templates. Ten microliters of PCR mixture, composed of 0.04 ng/μl template DNA, 0.5 pmol/μl primer pairs (Additional file 1), 1X PCR buffer (Bioline, UK), 0.2 mM dNTPs, 5 mM MgCl_2_, and 0.25 U BioTaq DNA polymerase (Bioline), was used. The thermal cycling conditions were as follows: a 1 min initial denaturation at 94 °C; 35 cycles of 30 s denaturation at 94 °C, 30 s of annealing at 60 °C, and a 90 s extension at 72 °C; and a 3 min final extension at 72 °C. The amplified DNAs were ligated into pGEM-T® Easy plasmids (Promega). Plasmids were introduced into *Escherichia coli* ElectroTen-blue (Stratagene, USA) by electroporation. Following the amplification of DNA inserts with the Illustra TempliPhi DNA Amplification Kit (GE Life Sciences, USA), nucleotide sequences were determined using the BigDye Terminator Kit (Applied Biosystems, USA) and an ABI 3730*xl* DNA sequencer (Applied Biosystems).

The transposon markers were designated as AhTE (*rachisypogaea*transposable element), as described in Shirasawa *et al.*[34].

### SSR markers derived from BAC-end sequences

BAC libraries for *A. duranensis* (AA) and *A. ipaënsis* (BB) have been constructed by Guimarães *et al.*[55]. Dr. Bertioli, University of Brasilia, Brazil, and his colleagues determined the end sequences of the BAC clones and designed primers on flanking regions of identified SSRs (Bertioli, personal communication). These BAC-end derived SSR markers kindly provided by Dr. Bertioli were also subjected to polymorphism analysis (Additional file 2).

### Polymorphism analysis of the DNA markers

In addition to the AHGS and AhTE markers developed in this study, a total of 4,449 previously published markers [11,15-20,34] were used for the polymorphism analysis (Additional file 2). PCR reactions were performed using 0.5 ng genomic DNA in each 5 μl reaction. In addition to template DNA, PCR reaction mixtures contained 1X PCR buffer (Bioline), 3 mM MgCl_2_, 0.04 U BIOTAQ^TM^ DNA polymerase (Bioline), 0.2 mM dNTPs, and 0.8 μM of each primer. The thermal cycling conditions were as follows: 1 min denaturation at 94 °C; 35 cycles of 30 s denaturation at 94 °C, 30 s of annealing at 60 °C, and a 1 min extension at 72 °C; and a final 3 min extension at 72 °C. The PCR products were separated by 10% polyacrylamide gel electrophoresis in 1X TBE buffer according to the standard protocol, or with a fluorescent fragment analyzer, ABI 3730*xl* (Applied Biosystems, USA). In the latter case, the data were analyzed using GeneMapper software (Applied Biosystems).

The previously reported SNP in *ahFAD2A* and transposon insertional polymorphism in *ahFAD2B* were also investigated to check the existence of polymorphisms in the NYF2 population [49,56,57]. The SNP was genotyped by the TaqMan assay with primer pairs (5’-CCCTTCACTCTTGTCTATTAGTTCCTTAT-3’ and 5’-TGATACCTTTGATTTTGGTTTTGG-3’) and TaqMan probes (FAM-labeled 5’-CCTCGACCGC**A**ACG-3’ for mutant allele and VIC-labeled 5’-CCTCGACCGC**G**ACG-3’ for the wild-type allele) on the 7900HT Fast Real-Time PCR System (Applied Biosystems). The TaqMan assay was performed according to the protocol of the TaqMan Genotyping Master Mix (Applied Biosystems). Transposon insertional polymorphisms were detected on 2% agarose gel as a difference in mobility of the DNA fragments that had been amplified by PCR with primers bF19: 5’-CAGAACCATTAGCTTTG-3’ and R1: 5’-CTCTGACTATGCATCAG-3’ [49]. PCR and electrophoresis were performed as described above.

### Construction of linkage maps

Linkage analysis was performed on segregated genotypic data from the two mapping populations using JoinMap® version 4 [58]. The marker loci were roughly classified using the JoinMap® grouping module with logarithm of odds (LOD) scores of 4.0–10.0. Marker order and genetic distance were calculated using a regression mapping algorithm with the following parameters: Haldane’s mapping function, recombination frequency ≤ 0.30, and LOD score ≥ 2.0. The graphical linkage maps were drawn with the MapChart program [59].

### Phenotyping and QTL analysis

A total of 15 morphological traits of the SKF2 population were investigated in the Peanut Plant Breeding Field of the Chiba Prefectural Agriculture and Forestry Research Center, Japan (35°37’54”N, 140°19’02”E). The seeds were sown in May, 2005 with 66 cm and 20 cm inter- and intra-row spacing, respectively. The flowering date for each plant was determined based on the opening of the first flower. Numbers and angles of branches, lengths of main stems and the longest branches, and fresh weights of the whole plant were measured at the harvesting stage. After drying of harvested pods under natural conditions for two weeks, the length, thickness, width, and weight of the matured pods were measured. In addition, constrictions on the pods were scored from 1 (deep) to 5 (shallow), and the shapes of the tips of the pods were also scored from 1 (round) to 5 (sharp). After that, numbers of seeds per plant and mean weights of single seeds were investigated. Colors of seed coats were classified as orange–yellow (2.5Y 8/6) or brown–red (2.5R 4/10), based on the Munsell color system.

To investigate the fatty acid content of the seeds, the 32 F_1_ parents of the NYF2 mapping population were planted in the Peanut Plant Breeding Field of the Chiba Prefectural Agriculture and Forestry Research Center in May, 2008 with 66 cm and 30 cm inter- and intra-row spacing, respectively. The F_2_ seeds were harvested in October and dried for one month in an open-air condition in their pods. One quarter of each of the dried seeds was cut off, and then 25 mg of the seeds was homogenized using TissueLyzer (Qiagen) with 400 μl of 100% (v/v) methanol. The homogenate was incubated at 70 °C for 15 min with 1,100 μl of 91% (v/v) methanol and then centrifuged at 15,000 rpm for 5 min. The supernatant was transferred to a glass tube. The pellet was resuspended in 750 μl of chloroform with 2 mg/ml nonadecanoic acid methyl ester (Sigma-Aldrich, USA), and the chloroform (resuspended pellet) layer was mixed with the supernatant layer. After washing the mixture with water, 900 μl chloroform and 1 ml of 3% (v/v) sulfuric acid in methanol were added to the chloroform layer, followed by washing with water. Then, 10 μl of the chloroform layer was dried with N_2_ gas, 70 μl n-heptane, 10 μl pyridine, and 10 μl N-methyl-N-(trimethylsilyl)trifluoroacetamide (Sigma-Aldrich). Fatty acid quantification was performed using gas chromatography time-of-flight mass spectrometry (GC-TOF-MS) with a 6890 N Network GC System (Agilent Technologies, USA) equipped with a column DB-17MS (length, 30 m; ID, 0.25 mm; film, 0.25 μm) (J&W Scientific, USA), coupled to Pegasus3 (Leco®, USA) with the following settings: inlet temperature, 250 °C; oven temperature, 70 °C for 5 min, increasing by 15 °C/min, and holding at 310 °C for 5 min; transfer tube temperature, 200 °C; and ion source temperature, 250 °C. Acquisition and analysis of mass spectral data were performed using the ChromaTOFTM version 2.32 optimized for Pegasus (Leco®). Concentrations of oleic acid, linoleic acid, palmitic acid, and stearic acid were estimated from calibration curves created using pure samples of each compound.

The phenotypic data regarding morphological traits of the SKF2 population were subjected to composite interval mapping by the Windows QTL Cartographer program [60]. The thresholds of LOD for each QTL were determined by 1,000 permutation tests. Since two genes, *ahFAD2A* and *ahFAD2B*, were reported to control the O/L ratio in seeds with epistatic interactions, QTL analysis of the NYF2 population was conducted with Genotype Matrix Mapping (GMM) software with the following parameters: Max Length of Locus Combination = 2; Min Number of Corresponding Samples = 1; Search Range = auto [61].

## Results

### Design of polymorphic genomic SSR markers

In the first library, a total of 11,673 genomic clones were sequenced. After removing redundant sequences, 2,661 primer pairs were designed to amplify the flanking regions of SSRs [DNA Data Bank of Japan (DDBJ): DH961577-DH964237] and designated as AHGS markers [62] (Additional file 3). Out of the 2,661 AHGS markers, 334 were screened for polymorphism compared with the four parental lines of the mapping populations using a fluorescent fragment analyzer. According to the results, 42 (12.6%) and 6 (1.8%) markers showed polymorphism between ‘Satonoka’ and ‘Kintoki’ and between ‘Nakateyutaka’ and ‘YI-0311’, respectively.

The second pair of genomic SSR libraries for *in silico* polymorphism analysis was constructed from ‘Satonoka’ and ‘Kintoki’, and a total of 7,872 and 8,208 clones were sequenced, respectively. After trimming vector, linker, and low-quality sequences, sequences with SSR motifs were assembled into 10,742 unique sequences consisting of 2,952 and 7,788 SSR-containing contigs and singlets, respectively. In the comparative analysis of the lengths of SSR motifs on each sequence, for 126 (4.3%) of the 2,952 contigs, the SSR repeats differed in length between ‘Satonoka’ and ‘Kintoki’, while 287 contigs (9.7%) had identical-length SSR repeats between the two lines. The remaining 2,539 contigs (86.0%), as well as 7,788 singlets, were composed of fragments derived from either line. After eliminating sequences that were identical to those from the first library, 126 primer pairs were designed to amplify polymorphic sequences, 287 were designed to amplify non-polymorphic sequences, and 3,606 additional, untested primer pairs were designed [63; DDBJ: DH964238-DH968256] (Additional file 3). Screening for polymorphism was performed using a fluorescent fragment analyzer with the parental lines of the mapping populations, the SKF2 and the NYF2, with 1,833 primer pairs consisting of 74, 121, and 1,638 primer pairs randomly selected from polymorphic, non-polymorphic, and untested SSR candidate data, respectively. A total of 582 of the tested 1,833 markers (31.8%) showed polymorphisms between ‘Satonoka’ and ‘Kintoki’, including 29 of the 74 candidate polymorphic (39.2%), 25 of the 121 candidate non-polymorphic (20.7%), and 528 of the 1,638 untested (32.2%) markers. Of the candidate polymorphic markers, the remaining 45 (60.8%) were monomorphic between the parental lines. Between ‘Nakateyutaka’ and ‘YI-0311’, 11 candidate polymorphic (14.9%), 10 candidate non-polymorphic (8.3%), and 162 candidate probable polymorphic (9.9%) markers showed polymorphism. As results of the polymorphism screenings with a total of 2167 genomic SSR markers, 624 (28.8%) and 189 (8.7%) showed polymorphisms between ‘Satonoka’ and ‘Kintoki’ and between ‘Nakateyutaka’ and ‘YI-0311’, respectively (Table 1).

**Table 1:** Polymorphic ratio of the investigated markers

	**Marker types**	**No. of tested markers**	**No. of polymorphic markers between 'Satonoka' and 'Kintoki'**	**%**	**No. of polymorphic markers between 'Nakateyutaka' and 'YI-0311'**	**%**	**References**
*DNA markers developed in this study*							
	Genomic SSRs	2167	624	28.8	189	8.7	This study
	Transposons	535	302	56.4	67	12.5	This study
*DNA markers derived from other studies*							
	Genomic SSRs	418	87	20.8	18	4.3	[16-20]
	EST-SSRs	3375	50	1.5	9	0.3	[11,15]
	BAC-end SSRs	152	25	16.4	6	3.9	Bertioli, pers. comm.
	Transposons	504	91	18.1	49	9.7	[34]
Total		7151	1179	16.5	338	4.7	

A total of 6,680 AHGS markers were designed from the first and second SSR-enriched genomic libraries. Out of these markers, the poly (CT)_n_ motif was the most abundant (2,390: 35.8%), followed by poly (AC)_n_ (1,496: 22.4%), due to the usage of biotinylated oligo probes of poly (AC)_12_ and poly (CT)_12_ in the construction of the libraries (Additional file 4). The frequencies of the other di-, tri-, and tetra-nucleotide repeat motifs were 13.1%, 5.6%, and 23.1%, respectively. The distributions of the SSR motifs found in the two libraries were not different. Between ‘Satonoka’ and ‘Kintoki’, the polymorphic ratio of the poly (CT)_n_ motif, 40.4% (498/1,232), was higher than that of the poly (AC)_n_ motifs, 14.2% (88/620) (Additional file 4).

### Design and polymorphism analysis of transposon markers

As with the AHGS marker development, transposon markers named AhTE were developed *via in silico* polymorphism analysis. A total of 13,248 and 12,351 clones derived from *AhMITE1*-enriched genomic libraries of ‘Satonoka’ and ‘Kintoki’, respectively, were sequenced. Of the total 25,599 sequences, 16,639 were identified as including *AhMITE1* sequences in the fragments. These were then assembled into 1,198 contigs. Cultivar-specific transposon insertions were identified in 511 out of the 1,198 contigs. In addition, 24 additional insertion sites were found from the libraries derived from the inverse PCR analysis. In total, 535 primer pairs were designed based on the flanking regions of identified *AhMITE1*s [63; DDBJ: DH968257-DH968767] (Additional file 5). When polymorphism analysis was performed using these 535 primer pairs with the four parental lines of the mapping population, a total of 302 (56.4%) and 67 (12.5%) markers exhibited polymorphism between ‘Satonoka’ and ‘Kintoki’, and between ‘Nakateyutaka’ and ‘YI-0311’, respectively (Table 1).

### Polymorphism analysis of the previously published marker loci

A total of 4,449 markers derived from other studies, including 418 genomic SSRs [16-20], 3,375 EST-SSRs [11,15], 152 BAC-end SSRs (Bertioli*,* personal communication), and 504 transposon markers [34], were used for polymorphism analysis between the four mapping parents (Additional file 2). Out of the 4,449 markers, 253 (5.7%), including 87 genomic SSRs (20.8%), 50 EST-SSRs (1.5%), 25 BAC-end SSRs (16.4%), and 91 transposon markers (18.1%), were selected as polymorphic markers between ‘Satonoka’ and ‘Kintoki’ (Table 1). Between ‘Nakateyutaka’ and ‘YI-0311’, 18 genomic SSRs (4.3%), 9 EST-SSRs (0.3%), 6 BAC-end SSRs (3.9%), and 49 transposon markers (9.7%) showed polymorphism (Table 1).

### Construction of the SKF2 genetic linkage map

**Figure 1:**
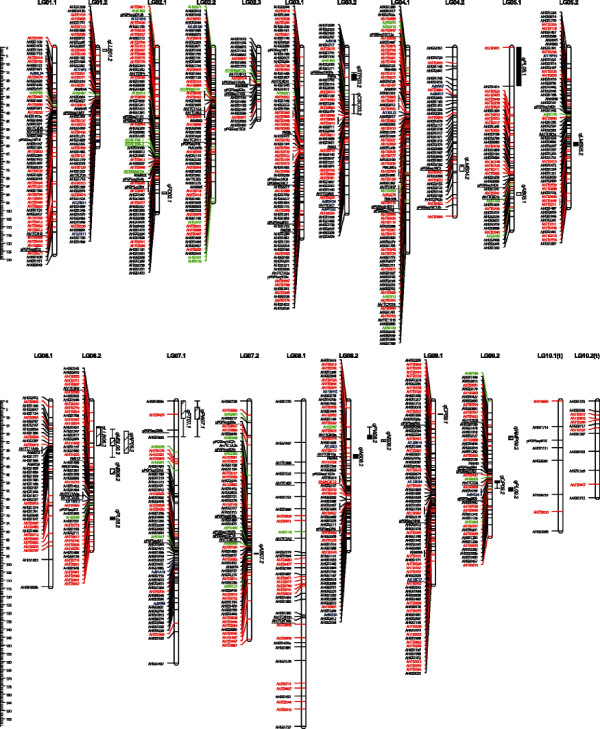
**The SKF2 linkage map and positions of QTLs for agronomical traits.** Scale bars on the left side describe map distance in centimorgans. Genomic SSR, transposon, EST-SSR, and BAC-end SSR markers are shown as black, red, green, and blue lines, respectively. Anchor markers to previously reported maps are underlined. QTLs with positive additive effects in Satonoka and Kintoki alleles are shown by hashed and black boxes, respectively, to the right of each linkage group. The vertical bars on the boxes show the regions over which significant LOD values were calculated by a permutations test (n = 1,000).

A total of 1,179 of the 7,151 (16.5%) tested markers showed polymorphism between ‘Satonoka’ and ‘Kintoki’. Nineteen, three, and one markers identified doubled, tripled, and quadrupled polymorphic loci, respectively, while 1,156 markers generated single polymorphic loci. Consequently, 1,207 segregating loci were generated from the 1,179 markers. A total of 1,114 of the 1,207 segregated loci (92.3%) were mapped onto 21 linkage groups (LGs), resulting in the SKF2 genetic linkage map. The length of the genetic linkage map was 2,166.4 cM, with distances ranging from 44.1 cM to 199.8 cM (Table 2, Figure 1, Additional file 6). Of the 1,114 loci, 949 were generated from markers developed in this study and in Shirasawa *et al.*[34].

**Table 2:** Length, number of mapped marker loci, and segregation distortion of the linkage maps

**Linkage group**	**SKF2**							**NYF2**						
	Length	Number of marker loci				Marker density	Segregation distortion ratio^a)^	Length	Number of marker loci				Marker density	Segregation distortion ratio^a)^
	(cM)	Total	AHGS	AhTE^b)^	Published^c)^	(cM/loci)	(%)	(cM)	Total	AHGS	AhTE^b)^	Published^c)^	(cM/loci)	(%)
LG01.1	126.9	59	29	23	7	2.2	28.8	74.7	46	28	13	5	1.7	4.3
LG01.2	60.9	61	34	19	8	1.0	19.7	56.9	13	10	3	0	4.7	15.4
LG02.1	101.3	69	36	20	13	1.5	18.8	66.6	30	22	7	1	2.3	3.3
LG02.2	94.7	66	34	18	14	1.5	28.8	4.9	2	2	0	0	4.9	50.0
LG02.3	44.1	24	14	3	7	1.9	16.7	21.1	5	3	1	1	5.3	0.0
LG03.1	117.0	78	42	26	10	1.5	39.7	97.2	31	19	9	3	3.2	0.0
LG03.2	101.7	55	32	14	9	1.9	16.4	76.0	9	6	1	2	9.5	0.0
LG04.1	125.6	87	34	37	16	1.5	31.0	104.3	13	6	7	0	8.7	23.1
LG04.2	103.5	40	29	7	4	2.7	32.5	106.6	15	7	7	1	7.6	20.0
LG05.1	112.2	43	21	18	4	2.7	9.3	62.4	6	4	2	0	12.5	33.3
LG05.2	98.3	60	37	16	7	1.7	23.3	84.2	8	1	7	0	12.0	0.0
LG06.1	114.0	42	27	12	3	2.8	38.1	-	-	-	-	-	-	-
LG06.2	91.7	56	25	24	7	1.7	19.6	146.5	44	21	17	6	3.4	9.1
LG07.1	160.9	55	38	9	8	3.0	29.1	167.3	44	29	10	5	3.9	6.8
LG07.2	146.3	62	26	21	15	2.4	43.5	2.9	2	1	1	0	2.9	0.0
LG08.1	199.8	38	17	15	6	5.4	15.8	98.5	24	8	12	4	4.3	4.2
LG08.2	91.8	68	38	20	10	1.4	57.4	49.4	15	10	4	1	3.5	13.3
LG09.1	112.6	81	48	27	6	1.4	19.8	72.8	7	2	4	1	12.1	14.3
LG09.2	83.1	50	25	15	10	1.7	22.0	16.7	10	5	2	3	1.9	0.0
LG10.1(t)	79.9	8	5	2	1	11.4	37.5	-	-	-	-	-	-	-
LG10.2(t)	59.3	12	7	5	0	5.4	8.3	-	-	-	-	-	-	-
LGX	-	-	-	-	-	-	-	23.9	2	2	0	0	23.9	0.0
Total	2166.4	1114	598	351	165	1.9	27.7	1332.9	326	186	107	33	4.3	7.7

a) Percentages of segregation loci with probability level P < 0.05.

b) Including the AhTE markers developed in the previous study (Shirasawa *et al.* 2012).

c) Including the BAC-end SSR markers developed by Bertioli *et al.* (unpublished).

The LGs were named according to the consensus numbers of the previously reported maps by comparison of the mapping position of the 122 commonly mapped markers [19,22,23,25,27,28,30] (Figure 1, Additional file 7). Eight homoeologous pairs of linkage groups (HGs) were identified, *i.e.*, 1, 3, 4, 5, 6, 7, 8, and 9. Those pairs were suffixed ‘.1’ and ‘.2’; for example, LG01.1 and LG01.2 (Table 2, Figure 1, Additional file 6). In HG2, three linkage groups were identified and designated as LG02.1, LG02.2, and LG02.3. Because two remaining linkage groups were generated with some common markers from previously reported maps, these linkage groups were tentatively named LG10.1(t) and LG10.2(t).

The average marker density of the SKF2 maps was 1.9 cM in total, ranging from 1.1 cM for LG01.2 to 11.4 cM for LG10.1(t) in the 21 linkage groups. The largest interval between two loci was 25.6 cM, observed between AHGS1270 and AHGS1947 on LG08.1. In the SKF2 map, 28.3% of the total marker loci showed segregation distortions, ranging from 42.2% in LG07.2 to 8.3% in LG10.2(t) (Table 2, Additional file 6).

### Construction of the NYF2 linkage map

A total of 338 of the 7,151 (4.7%) tested markers showed polymorphism between ‘Nakateyutaka’ and ‘YI-0311’. While four markers identified double polymorphic loci, the other 334 markers generated single polymorphic loci. Consequently, 342 polymorphic loci were generated from the 338 markers. Because the SNP for the D150N change in *ahFAD2A*[56,57] and the transposon insertion polymorphism at the 665^th^ base in *ahFAD2B*[49] segregated in the NYF2 population, the *ahFAD2A* and *ahFAD2B* genes were subjected to genotyping of the F_2_ population together with the 342 polymorphic loci. A total of 326 segregating loci (94.8%) formed 19 linkage groups (LGs) (Table 2, Additional file 6, Additional file 8). Of the 19 LGs, 18 were numbered corresponding to the SKF2 maps and the previously reported maps with 18 anchor markers [19,22,23,25,27,28,30] (Additional file 7, Additional file 8). The remaining linkage group was tentatively named LGX because this LG had the possibility of corresponding to either LG06.1, LG10.1(t), or LG10.2(t) of the SKF2. The *ahFAD2A* and *ahFAD2B* genes were mapped onto LG9.2 and LG9.1, respectively, as reported by Qin *et al.*[30]. The total length of the map was 1,332.9 cM, with individual LGs ranging from 2.9 cM (LG07.2) to 167.3 cM (LG07.1). The average marker density was 4.3 cM, ranging from 1.7 cM in the LG01.1 to 23.9 cM in the LGX. In the NYF2 map, 7.7% of the marker loci showed significant segregation distortions. The highest segregation distortion was observed in LG2.2 (50%), whereas seven LGs had no distorted loci (Table 2, Additional file 6). Of the 326 loci, 293 were generated from markers developed in this study and in Shirasawa *et al.*[34].

### Detection of QTLs for agronomical traits

Phenotypic values of 15 morphological quantitative traits investigated in SKF2 exhibited transgressive segregation relative to the mapping parents (Additional file 9). A total of 23 significant QTLs were detected for the 15 investigated traits (Table 3, Figure 1). The phenotype variance explained by the QTLs ranged from 4.8 to 28.2%. A QTL for flowering date was detected on LG02.1 and named as *qFD02.1*, of which the ‘Kinotki’ allele had an effect of late flowering. Two QTLs for branching angle, *qAB05.1* and *qAB07.2*, were detected on LG05.1 and LG07.2, with opposite directions of additive effects. As traits for plant size, three (*qLMS06.2*, *qLMS04.2*, and *qLMS05.2*), two (*qLLB06.2* and *qLLB01.2*), one (*qNB06.2*), and one (*qWP06.2*) QTLs affecting the length of the main stem, length of the longest branch, number of branches, and weight of the plant were detected, respectively, all of which had positive additive effects for the ‘Kintoki’ allele except for *qLMS05.2*. QTLs for length of the main stem, length of the longest branch, and weight per plant were detected in the same marker interval in LG06.2. The traits showed significant correlation with each other, and therefore it was suggested that the identified QTLs controlled plant biomass (Additional file 10). For pod characters, three QTLs for length (*qPL05.1*, *qPL09.2*, and *qPL06.2*), one for thickness (*qPT07.1*), two for wideness (*qPW07.1* and *qPW08.2*), two for constriction (*qCP09.2* and *qCP09.1*), and one for shape of the beak (*qSTP03.2*) were detected. QTLs for seed weight (*qWS08.2*) and number of seeds per plant (*qNS08.2*) did not overlap but did map to the same LG (LG08.2). A QTL cluster was found for pod thickness and width on LG07.1, and the correlation was significant (Additional file 10). Thus, the QTL was considered to regulate lateral growth of pods. Other QTLs related to pod character, *i.e.*, weight of mature pod per plant (*qWMP09.2*), length of pod (*qPL09.2*), and constriction of pod (*qCP09.2*), mapped on LG09.2 but in different marker intervals. The trait for red seed coat color (*qCSC03.2*), which is not a quantitative but a qualitative trait, segregated into 77 red and 17 orange–yellow offspring, fitting a 3:1 ratio by the chi-square test (*χ*^2^ = 0.12, *p* = 0.73). This trait mapped to a single locus between two marker loci, PM3a and AHGS1792, in the LG03.2 with an LOD value of 67.4.

**Table 3:** Positions, effects, and phenotypic variation explained by QTLs for 15 agronomic traits detected in the SKF2 population

**Trait**	**QTL name**	**Linkage group**	**Marker interval**	**LOD**	**Additive effect^a)^**	**Dominant effect^a)^**	**Phenotypic variation explained (%)**
Flowering date (day)	*qFD02.1*	LG02.1	AHGS2736-AHGS1251	5.0	1.7	−2.4	19.5
Angle of branch (1: erect to 5: spread-out)	*qAB05.1*	LG05.1	AHGS2534-AHGS2622	5.7	0.5	−0.9	11.9
	*qAB07.2*	LG07.2	AHGS1215-AhTE0615	4.6	−0.6	0.2	23.2
Length of main stem (cm)	*qLMS06.2*	LG06.2	AhTE0589-Ah1TC3H7	7.9	2.8	7.2	4.8
	*qLMS04.2*	LG04.2	AHGS2155-AHGS3725	6.8	4.5	2.3	19.2
	*qLMS05.2*	LG05.2	AHGS2020-AHGS2450	4.6	−4.4	2.0	15.7
Length of the longest branch (cm)	*qLLB06.2*	LG06.2	AhTE0697-Ah1TC3H7	5.7	7.6	9.9	21.1
	*qLLB01.2*	LG01.2	AHGS1813b-AhTE1016	4.7	4.9	−7.0	14.2
Number of branches (branch)	*qNB06.2*	LG06.2	AhTE0967-AhTE0074	5.1	5.2	3.8	15.6
Weight of plant (g)	*qWP06.2*	LG06.2	AhTE0697-Ah1TC3H7	5.4	7.8	12.9	11.8
Weight of mature pod per a plant (g)	*qWMP09.2*	LG09.2	AHGS0422-AHGS2635	7.2	5.4	2.2	28.1
Length of pod (mm)	*qPL05.1*	LG05.1	AhTE0601-AHGS1413	6.7	−2.4	2.2	28.2
	*qPL09.2*	LG09.2	AHS1684-AhTE0707	5.4	−1.5	−1.6	8.4
	*qPL06.2*	LG06.2	AhTE0745-AhTE0826	4.9	−2.1	1.1	20.5
Thickness of pod (mm)	*qPT07.1*	LG07.1	AHGS1803a-AhTE0025	11.9	1.0	0.4	21.7
Width of pod (mm)	*qPW07.1*	LG07.1	AhTE0025-pPGPSeq2E6b	11.4	0.6	0.4	15.2
	*qPW08.2*	LG08.2	AHGS1286-AHGS2249	9.5	−0.7	0.4	25.5
Constriction of pod (1: deep to 5: shallow)	*qCP09.2*	LG09.2	AHGS0362-AhTE0726	8.1	−0.6	−0.5	18.1
	*qCP09.1*	LG09.1	AHGS1673-AhTE0222	6.2	0.3	0.6	6.9
Shape of tip of pods (1: round to 5: sharp)	*qSTP03.2*	LG03.2	AhTE0570-AHGS1744	5.9	−0.3	0.7	9.9
Weight of a seed (g)	*qWS08.2*	LG08.2	AhTE0846-AhTE0974	4.9	−0.1	0.1	19.1
Number of seeds per a plant (grain)	*qNS08.2*	LG08.2	AHGS1286-AHGS2249	5.2	3.0	28.3	6.8
Color of seed coat (2: orange–yellow to 5: brown–red)	*qCSC03.2*	LG03.2	PM3a-AHGS1792	67.4	0.6	1.4	9.7

a) Effect of 'Kintoki' allele.

### Relationship between mutations of *ahFAD2* genes and the O/L ratio

**Figure 2:**
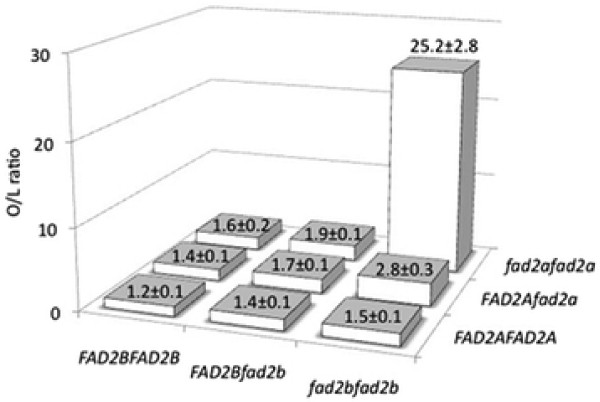
**Ratio of oleic-acid to linoleic-acid content (O/L ratio) in peanut seeds of different genotypes of*ahFAD2A*and*ahFAD2B*genes in 185 NYF2 plants.** Dominant and recessive alleles of *FAD2* genes were shown by capital (*FAD2A*, *FAD2B*) and lowercase (*fad2a*, *fad2b*) letters, respectively. Numeric descriptions are average values for the O/L ratio in peanut seeds, with standard errors.

In NYF2, fatty acid concentration was determined for the parental and 185 individual F_2_ seeds. The O/L ratio of the parental lines, ‘Nakateyutaka’ and ‘YI-0311’, were 0.98 ± 0.11 and 49.4 ± 4.9, respectively. The O/L ratio of the F_2_ lines ranged from 0 to 35.2. Of the 185 F_2_ plants, the O/L ratios of the 178 lines were as low as that of the ‘Nakateyutaka’, while the other seven were, remarkably, as high as that of ‘YI-0311’. The O/L ratio did not significantly correlate with the sum of oleic-acid and linoleic-acid contents, or oleic-acid content, but showed negative correlation with linoleic-acid content (data not shown). This result suggests that a change in the O/L ratio could mainly be attributed to linoleic-acid biosynthesis activity. The significant association between O/L ratio in seeds and the combination of genotypes of *ahFAD2A* and *ahFAD2B* was confirmed by GMM analysis with F value = 1,619.7, *p* < 0.01. The phenotype variance explained by these two genes was 89.7%. All seven of the F_2_ seeds that showed a high O/L ratio exhibited homozygous genotypes derived from ‘YI-0311’ on the *ahFAD2A* and *ahFAD2* genes (Figure 2).

## Discussion

In this study, we developed a high-density genetic linkage map, SKF2, of a total length of 2,166.4 cM consisting of 1,114 marker loci (Figure 1, Table 2, Additional file 6). Genetic linkage maps in *Arachis* spp. have been constructed using mapping populations derived from crosses between interspecific diploids [14,19,22,23] or synthetic tetraploids [13,27], as well as cultivated tetraploids [21,24-26]. In addition, the integration of more than two maps by connecting common markers as anchors has been conducted to produce a higher number of marker loci than that on single maps [28-31]. While it is true that map integration is an effective way to increase marker loci on a single map, the development of new markers is still required to saturate linkage maps in peanut. As far as we know, the SKF2 map covering 2,166.4 cM with 1,114 loci is the highest-density genetic linkage map in *Arachis*, and probably covers a large portion of the peanut genome because the total length of the map is almost equal to those of maps for tetraploids (2,210 cM with 370 loci [13], 1,844 cM with 298 loci [27], and 1,785 cM with 191 loci [26]), and double those of maps for wild diploids (1,063 cM with 117 loci [14], 1,231 cM with 170 loci [19], and 1,294 cM with 149 loci [23]).

Our results suggested that *in silico* polymorphism analysis worked effectively for the development of polymorphic SSR and transposon markers. This was the first time *in silico* polymorphism analysis has been used in peanut. The polymorphic ratios in SKF2 increased from 15.9% (=133/838) to 54.4% (=331/609) in total, *i.e.*, 12.6% (=42/334) to 39.2% (=29/74) for genomic SSR markers and 18.1% (=91/504) to 56.4% (=302/535) for transposon markers, by employing *in silico* polymorphism analysis. In this study, we performed empirical analysis for 1,833 of 4,019 primer pairs generated *via in silico* polymorphism analysis. If 32% of SSR markers derived from a second library show polymorphisms in the SKF2 population, an additional 700 [=0.32 × (4019–1833)] markers would map to the SKF2 map.

Though *in silico* polymorphism analysis was performed for parental lines of SKF2, the analysis increased polymorphic ratios in the NYF2 population as well. This result suggested that *in silico* polymorphism analysis between two lines enhances the efficiency of polymorphic marker development in this species. Koilkonda *et al.*[11] investigated genetic distances for 16 *Arachis* spp. accessions, including the four parental lines used in this study. According to their results, greater genetic diversity was observed among cultivated peanut lines than among our four parental lines. Thus, we considered that marker subsets developed in this study could be useful sources for obtaining polymorphic markers in other mapping populations. However, in parallel, the generation of an insufficient number of polymorphic markers in NYF2 suggested that additional *in silico* polymorphism analysis is required to develop polymorphic markers that can differentiate between closely-related lines such as the parents of NYF2.

Meanwhile, of the candidate polymorphic sequences, 60.8% of the SSRs and 43.6% of the transposon markers did not show polymorphisms. Two reasons might account for such identification of false positives in *in silico* polymorphism analysis. The first is related to the presence of the A and B genomes in tetraploid peanut. For example, in the case of sequences of one parent being derived from only the A genome and those of another parent being derived from only the B genome, there is a high possibility that homoeologous polymorphisms could be identified but not allelic polymorphisms. Another possible reason is sequencing errors introduced through the use of the Sanger method. New robust sequencing technologies, *e.g.*, pyrosequencing and sequencing by synthesis or ligation, which have been used in massive parallel sequencers, may overcome these two possible causes because the principles underlying the sequencing reaction are different from those of the Sanger method, and duplication ratios of sequences per target region can be increased because of the ability to conduct high-throughput data generation.

The number of linkage groups of the SKF2 was one more than the number of haploid chromosomes of *A. hypogaea*, and the diversified density of DNA markers on each linkage group ranged from 1.1 to 11.4 cM/marker-locus. This indicates that genetic diversity is different between chromosomes of cultivated peanut. The polymorphism analysis in diploid *Arachis* species suggested that genetic diversity between B genome species was considerably lower than that between A genome species [23]. Though we cannot draw any conclusions from this study, it was predicted that similar differences might occur in the tetraploid genome. It has been suggested that the *AhMITE1*s originated from the B genome [34] but are currently distributed throughout the whole peanut genome (Figure 1, Table 2, Additional file 6, Additional file 8). This indicates that the *AhMITE1*s transposed from the B genome to the combined A and B genome without any bias in insertional position.

In the present study, agronomically important traits for flowering date, plant architecture, pod and seed characters, and seed quality were identified. Whereas several QTLs for drought stress tolerance and resistance to rust and foliar diseases have been reported in peanut [24-26,28,31], QTL analyses focused on morphological and physiological traits considered important for breeding have not been conducted. On the other hand, genomic and genetic studies of such traits have progressed in soybean [63-66] and *L. japonicus*[67]; both of these genomes have been sequenced [5,6]. If the genetic knowledge gained through comparative genomics using models is to be applied to crop legumes, the *in silico* polymorphism analysis must be effective for EST-SSR markers. Because nucleotide sequences of ESTs generally show higher levels of similarity across different species, genera, and families than sequences from intergenic regions, generating and mapping additional EST-SSR markers might help the progress of comparative analysis with other *Arachis* spp. and model legumes. Further genetic analysis will provide helpful information that will allow the identification of QTLs in peanut corresponding to the genome sequences of model legumes.

Candidate gene approaches, as well as comparative maps, will greatly help to develop DNA markers tightly linked to important traits that have been gained through the study of model legumes. Direct selection of two recessive alleles of the *FAD2* genes will facilitate high oleic-acid peanut breeding. Furthermore, introgression breeding of the high-oleic acid trait into elite cultivars can be easily performed with recurrent backcrossings and marker-assisted selection for the sake of monitoring both alleles of the *FAD2* genes along with genetic background nature. Similarly, flowering date, pod and seed characters, and plant architecture, as well as stress tolerance and disease resistance, can be efficiently altered by molecular breeding with marker-assisted selection.

## Conclusion

The efficiency of polymorphic marker development was improved remarkably by using *in silico* polymorphism analysis, in comparison with the previous method, in which primers were simply designed based on the flanking sequences of SSR motifs. The resultant linkage maps possess the highest number of marker loci in cultivated peanut as well as *Arachis* spp. Moreover, the developed linkage maps are applicable to the identification of QTLs and genes for agronomical traits, including seed quality. These data should be useful for genetics, genomics, and breeding in *Arachis* spp. This type of *in silico* polymorphism analysis should also be applicable to other crop species.

## Abbreviations

BAC: Bacterial artificial chromosome; cDNA: Complementary DNA; EST: Expressed sequence tag; GC-TOF-MS: Gas chromatography time-of-flight mass spectrometry; GMM: Genotype Matrix Mapping; HG: Homoeologous linkage groups; LG: Linkage group; LOD: Logarithm of odds; PCR: Polymerase chain reaction; QTL: Quantitative trait locus; SSR: Simple sequence repeat; SNP: Single nucleotide polymorphism.

## Authors’ contributions

KS, SI, and ST designed this work. CK, HK, MH, and S Suzuki established the mapping populations and performed phenotypic analysis of the plant materials. TK and YN provided their developed SSR markers. AK, AW, CT, HT, KK, KS, M Kato, MW, PK, S Sasamoto, WT, and YK performed the molecular experiments. AN, HH, KA, KS, PK, and SI analyzed the data. HH and M Kohara constructed the database. KA, KS, and SI wrote the manuscript. All authors read and approved the final manuscript.

## Supplementary Material

Additional file 1: **Table S1.** Primer combinations used for the inverse PCR. Click here for file

Additional file 2: **Table S2.** Primer sequences for the published EST and genome-derived SSR and transposon markers and BAC-end SSR markers derived from Bertioli *et al.* (unpublished) and used in this study. Click here for file

Additional file 3: **Table S3.** Primer sequences and SSR motifs of genomic SSR markers. Click here for file

Additional file 4: **Table S4.** Number of SSR motifs and polymorphic markers in the genomic SSR markers. Click here for file

Additional file 5: **Table S5.** Primer sequences of transposon markers. Click here for file

Additional file 6: **Table S6.** Linkage maps of SKF2 and NYF2. Click here for file

Additional file 7: **Table S7.** Positions of SSR markers commonly included in previously reported maps. Click here for file

Additional file 8: The NYF2 linkage map. Scale bars on the left side describe the map distance in centimorgans. Genomic SSR, transposon, EST-SSR, and BAC-end SSR markers are shown as black, red, green, and blue lines, respectively. Anchor markers to previously reported maps are underlined. The *ahFAD2* genes are shown in italics. Click here for file

Additional file 9: **Figure S1.** Distributions of the investigated traits. Click here for file

Additional file 10: **Table S8.** Correlation coefficient between quantitative traits. Click here for file
