# Supplementary figures and images for: In silico polymorphism analysis for the development of simple sequence repeat and transposon markers and construction of linkage map in cultivated peanut
Source: BMC Plant Biol. 2012 Jun 6;12:80. doi: 10.1186/1471-2229-12-80 (PMC3404960; doi:10.1186/1471-2229-12-80)

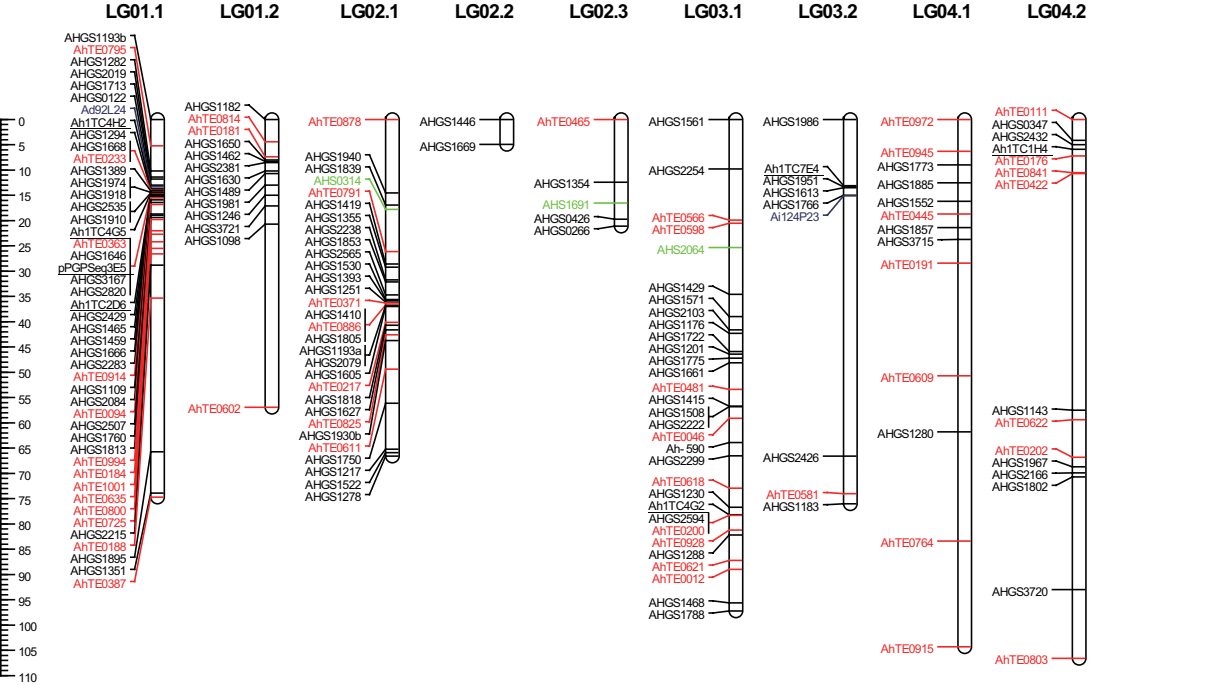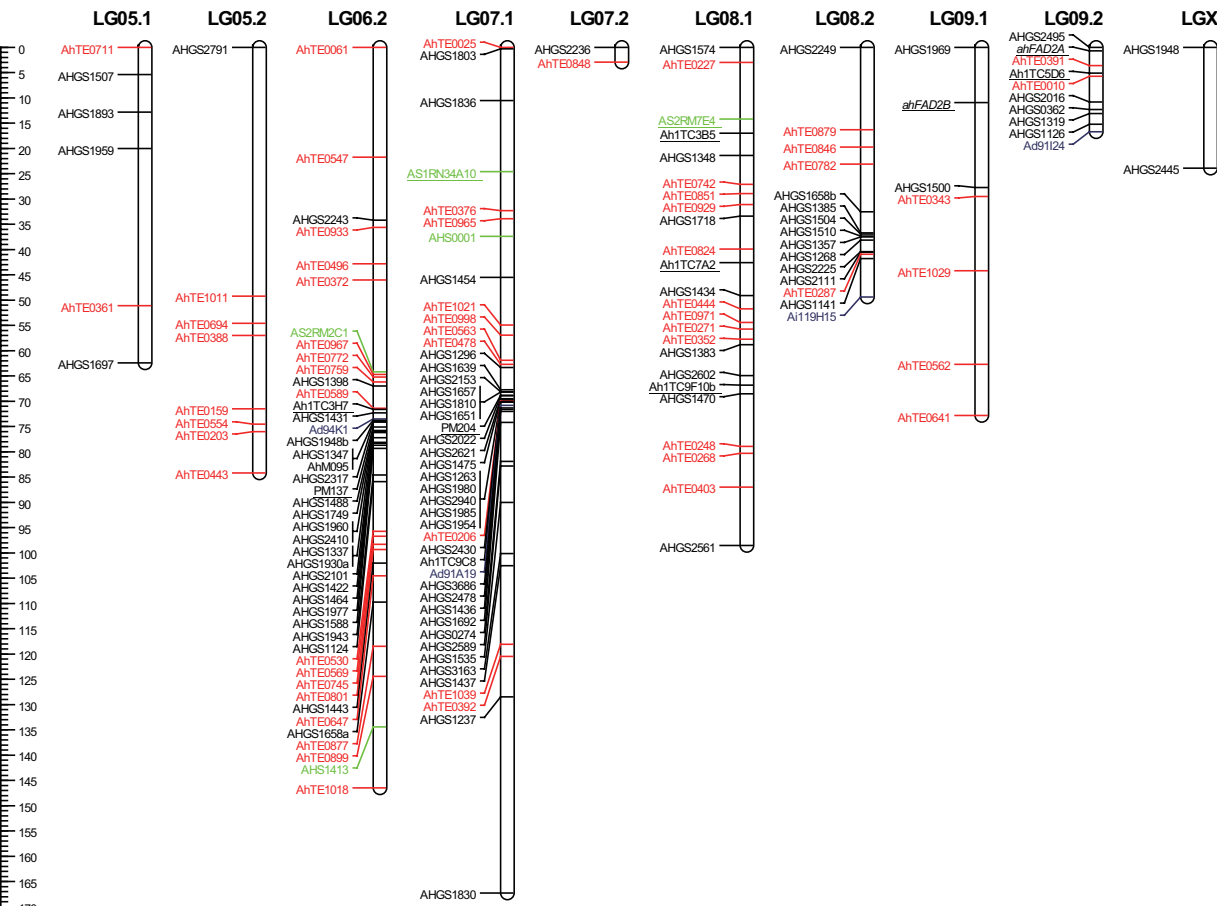

Supplement: Additional file 8: — The NYF2 linkage map. Scale bars on the left side describe the map distance in centimorgans. Genomic SSR, transposon, EST-SSR, and BAC-end SSR markers are shown as black, red, green, and blue lines, respectively. Anchor markers to previously reported maps are underlined. The ahFAD2 genes are shown in italics. [file 1471-2229-12-80-S8.pdf]
