# Supplementary material for: In silico polymorphism analysis for the development of simple sequence repeat and transposon markers and construction of linkage map in cultivated peanut
Source: BMC Plant Biol. 2012 Jun 6;12:80. doi: 10.1186/1471-2229-12-80 (PMC3404960; doi:10.1186/1471-2229-12-80)
Supplement: Additional file 9: — Figure S1. Distributions of the investigated traits. [file 1471-2229-12-80-S9.pdf]

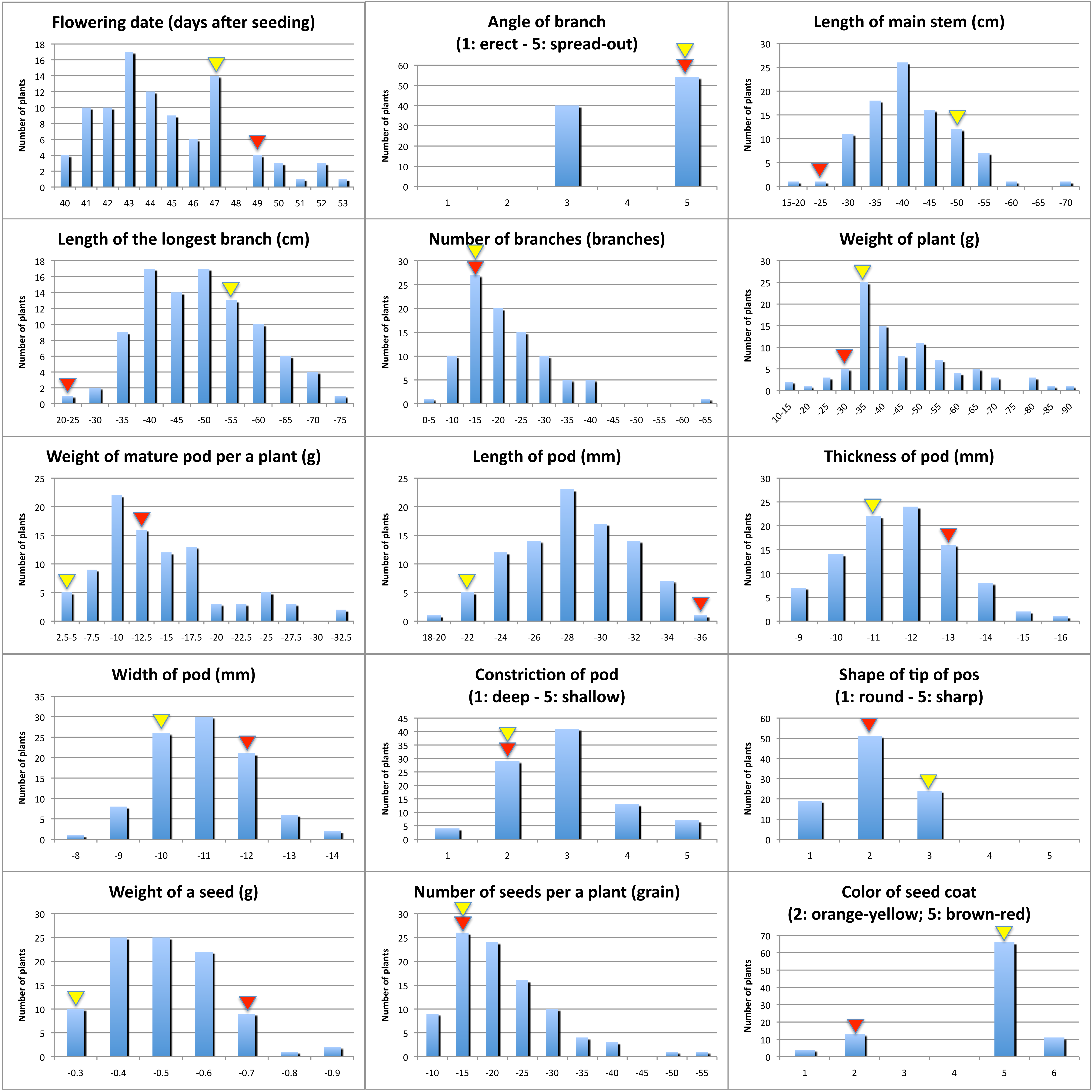

Supplementary Figure S1 Distributions of the investigated traits.
Classes of 'Satonoka' and 'Kintoki' are shown by red and yellow triangles, respectively.
